# Quantitative Dynamic-Enhanced MRI and Intravoxel Incoherent Motion Diffusion−Weighted Imaging for Prediction of the Pathological Response to Neoadjuvant Chemotherapy and the Prognosis in Locally Advanced Gastric Cancer

**DOI:** 10.3389/fonc.2022.841460

**Published:** 2022-03-29

**Authors:** Yongjian Zhu, Zhichao Jiang, Bingzhi Wang, Ying Li, Jun Jiang, Yuxin Zhong, Sicong Wang, Liming Jiang

**Affiliations:** ^1^Department of Diagnostic Radiology, National Cancer Center/National Clinical Research Center for Cancer/Cancer Hospital, Chinese Academy of Medical Sciences and Peking Union Medical College, Beijing, China; ^2^Department of Medical Oncology, National Cancer Center/National Clinical Research Center for Cancer/Cancer Hospital, Chinese Academy of Medical Sciences and Peking Union Medical College, Beijing, China; ^3^Department of Pathology, National Cancer Center/National Clinical Research Center for Cancer/Cancer Hospital, Chinese Academy of Medical Sciences and Peking Union Medical College, Beijing, China; ^4^Department of Pancreatic and Gastric Surgery, National Cancer Center/National Clinical Research Center for Cancer/Cancer Hospital, Chinese Academy of Medical Sciences and Peking Union Medical College, Beijing, China; ^5^Pharmaceutical Diagnostic Team, GE Healthcare, Life Sciences, Beijing, China

**Keywords:** gastric cancer, neoadjuvant chemotherapy, magnetic resonance imaging, intravoxel incoherent motion diffusion-weighted imaging, dynamic contrast-enhanced magnetic resonance imaging, tumor regression grade, response prediction, prognosis

## Abstract

**Background:**

This study aimed to explore the predictive value of quantitative dynamic contrast-enhanced MRI (DCE-MRI) and intravoxel incoherent motion diffusion-weighted imaging (IVIM-DWI) quantitative parameters for the response to neoadjuvant chemotherapy (NCT) in locally advanced gastric cancer (LAGC) patients, and the relationship between the prediction results and patients’ prognosis, so as to provide a basis for clinical individualized precision treatment.

**Methods:**

One hundred twenty-nine newly diagnosed LAGC patients who underwent IVIM-DWI and DCE-MRI pretreatment were enrolled in this study. Pathological tumor regression grade (TRG) served as the reference standard of NCT response evaluation. The differences in DCE-MRI and IVIM-DWI parameters between pathological responders (pR) and pathological non-responders (pNR) groups were analyzed. Univariate and multivariate logistic regressions were used to identify independent predictive parameters for NCT response. Prediction models were built with statistically significant quantitative parameters and their combinations. The performance of these quantitative parameters and models was evaluated using receiver operating characteristic (ROC) analysis. Clinicopathological variables, DCE-MRI and IVIM-DWI derived parameters, as well as the prediction model were analyzed in relation to 2-year recurrence-free survival (RFS) by using Cox proportional hazards model. RFS was compared using the Kaplan–Meier method and the log-rank test.

**Results:**

Sixty-nine patients were classified as pR and 60 were pNR. K^trans^, k_ep_, and v_e_ values in the pR group were significantly higher, while ADC_standard_ and D values were significantly lower than those in the pNR group. Multivariate logistic regression analysis demonstrated that K^trans^, k_ep_, v_e_, and D values were independent predictors for NCT response. The combined predictive model, which consisted of DCE-MRI and IVIM-DWI, showed the best prediction performance with an area under the curve (AUC) of 0.922. Multivariate Cox regression analysis showed that ypStage III and NCT response predicted by the IVIM-DWI model were independent predictors of poor RFS. The IVIM-DWI model could significantly stratify median RFS (52 vs. 15 months) and 2-year RFS rate (72.3% vs. 21.8%) of LAGC.

**Conclusion:**

Pretreatment DCE-MRI quantitative parameters K^trans^, k_ep_, v_e_, and IVIM-DWI parameter D value were independent predictors of NCT response for LAGC patients. The regression model based on baseline DCE-MRI, IVIM-DWI, and their combination could help RFS stratification of LAGC patients.

## Introduction

Gastric cancer (GC) represents the fifth most frequent cancer worldwide with 1,089,103 new cases (5.6%) and the fourth leading cause of cancer-related death (7.7%) with 768,793 deaths each year in 2020, according to global cancer statistics (1). In particular, gastric cancer has a high incidence in East Asia, which accounts for about 60% newly diagnosed cases worldwide (1, 2).

In China, about 70%–80% of GC patients were staged as locally advanced gastric cancer (LAGC) at the time of diagnosis (3), which was defined as a tumor invading the muscularis propria or deeper layer of the gastric wall without distant metastasis, often with a high rate of lymph node metastasis and poor clinical prognosis. The current treatment strategy for LAGC includes radical surgical resection through a multidisciplinary team (MDT) discussion, but the recurrence rate after radical resection is still up to 40%–60%, and the overall 5-year survival rate is only 20%–40% (4, 5). Several large international clinical trials (MAGIC and FFCD trials) showed that neoadjuvant chemotherapy (NCT) could significantly improve the R0 resection rate of LAGC patients, and the 5-year overall survival (OS) rate could be increased 10%–15% compared with the surgery alone group (6). NCT has been recognized as the standard treatment strategy for LAGC based on the National Comprehensive Cancer Network (NCCN) and Chinese Society of Clinical Oncology (CSCO) guidelines for gastric cancer (7, 8).

However, the therapeutic response of LAGC to NCT is highly heterogeneous, and the prognosis of patients who have good responses is significantly better than that of patients with poor responses (9). Patients with poor treatment response could not benefit from NCT, and NCT might increase treatment-related adverse reactions and medical cost, delay the optimal timing of surgery, or lead to tumor progression, resulting in poor prognosis (10). Currently, tumor regression grade (TRG) is widely used as an objective indicator for evaluating the NCT response in LAGC (11), but it can only be obtained through postoperative pathological examination. Therefore, an accurate prediction of the response to NCT in LAGC patients before surgery would be of great clinical significance, through which could screen patients who might benefit from NCT and further make an appropriate and personalized treatment plan.

Quantitative dynamic contrast-enhanced MRI (DCE-MRI) and intravoxel incoherent motion diffusion-weighted imaging (IVIM-DWI) are commonly used as functional MRI imaging techniques. DCE-MRI could obtain quantitative parameters of hemodynamics non-invasively through the pharmacokinetic model (12). IVIM-DWI proposed by Le Bihan et al. (13) used a biexponential model with multiple *b* values to obtain multiple parameters, which can distinguish pure molecular diffusion and microcirculatory perfusion in the capillary networks, compared with conventional DWI (14). Previous studies have found that quantitative parameters of DCE-MRI and IVIM-DWI can be used as an imaging biomarker of clinical, histopathological, and prognostic factors in different tumors (15–17). However, due to respiratory movement and gastrointestinal motility, these functional MRI techniques are rarely used in gastric research. There have been also no reports on the prediction of NCT response and prognosis in LAGC using DCE-MRI and IVIM-DWI.

Therefore, the purpose of this study is to explore the predictive value of DCE-MRI and IVIM-DWI quantitative parameters for the pathological treatment response of NCT in LAGC patients, and the relationship between the prediction results and patient prognosis, to provide a basis for clinical individualized precision treatment.

## Materials and Methods

This prospective study was conducted in accordance with the Declaration of Helsinki and approved by the Independent Ethics Committee of the Cancer Hospital, Chinese Academy of Medical Sciences (Beijing, China), and written informed consent was acquired from each subject before inclusion.

### Patients

A total of 167 consecutive patients with newly diagnosed resectable LAGC who underwent gastric MRI in National Cancer Center/Cancer Hospital, Chinese Academy of Medical Sciences, and Peking Union Medical College from January 2016 to December 2018 were initially enrolled. Patients were included according to the following criteria: 1) pathologically confirmed gastric adenocarcinoma on gastroscopy; 2) no contraindication to MR examinations; 3) locally advanced stage (cT3-4aN1-3M0) according to the American Joint Committee on Cancer (AJCC) TNM staging system (8th edition) (18) as determined by pretreatment CT, MRI, or endoscopic ultrasonography; 4) no previous treatment before MRI examination; 5) NCT performed within 1 week after MR examination; 6) R0 radical gastrectomy within 30 days after the completion of NCT; and 7) regular follow-up after surgery.

**Figure 1:**
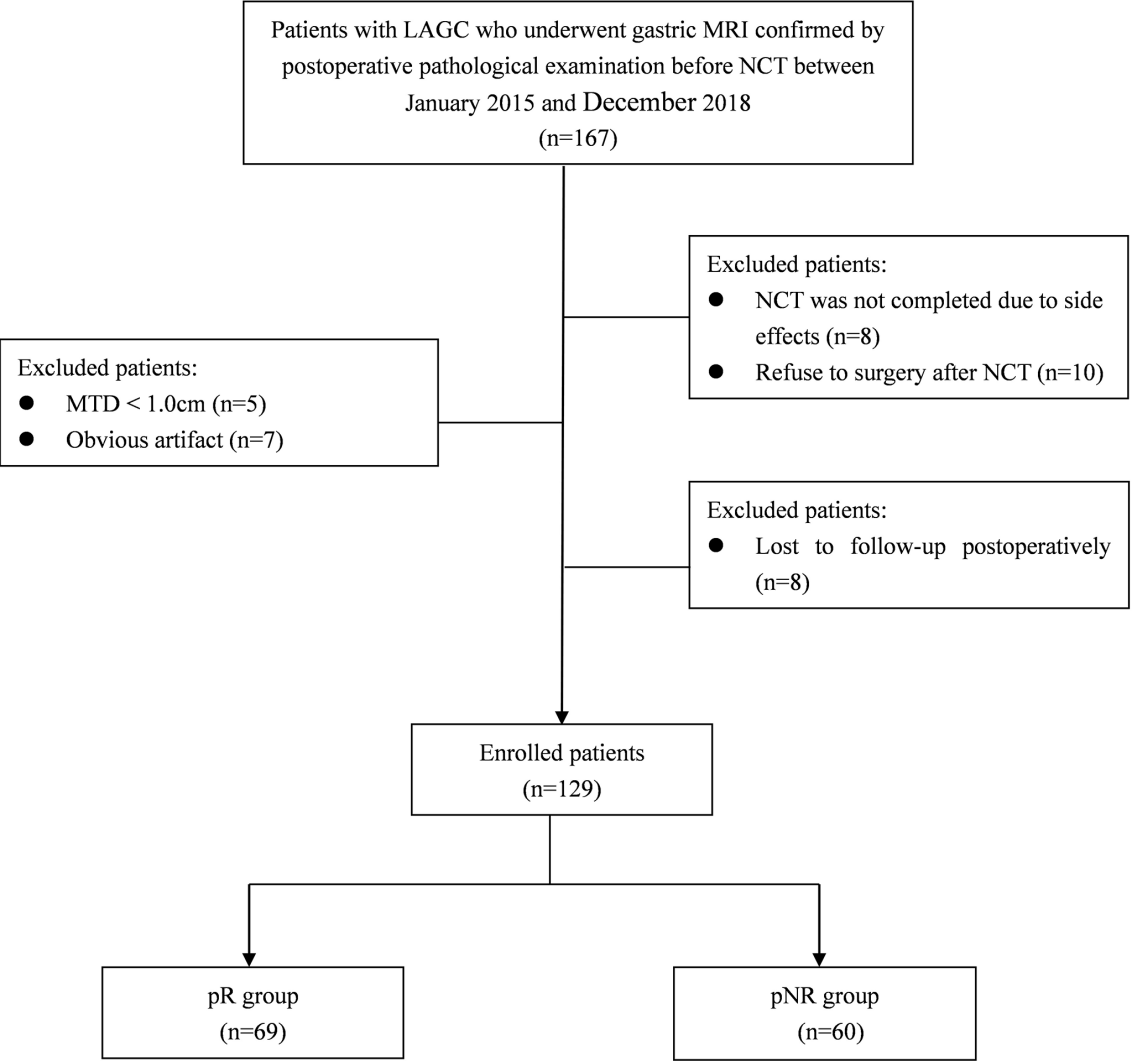
Diagram showing the recruitment of the study population and exclusion criteria. LAGC, locally advanced gastric cancer; NCT, neoadjuvant chemotherapy; MTD, maximum tumor diameter; pR, pathological responders; pNR, pathological non-responders.

The enrolment flowchart of the study cohort is summarized in **Figure 1**. Thirty-eight patients were excluded for the following reasons: 1) low quality of MR images due to obvious respiratory movement or gastrointestinal motility artifact or significant image distortion (n = 7); 2) maximum tumor diameter (MTD) <1.0 cm (n = 5); 3) NCT not completed due to severe adverse reactions (n = 8); 4) refused surgery after complete NCT (n = 10); and 5) lost to follow-up postoperatively (n = 8). Finally, a total of 129 patients were included in this study, including 107 men and 22 women, with a median age of 60 years (range from 28 to 76 years).

### MRI Data Acquisitions

Patients were asked to fast for 6–8 h prior to MR examinations to empty the gastrointestinal tract and underwent breath-holding training. In order to avoid the artifact of gastrointestinal peristalsis, patients without contraindications (i.e., glaucoma, prostate hypertrophy, asthma, or severe heart disease) were injected with 10 mg of anisodamine hydrobromide (Hangzhou Minsheng Pharmaceutical Co., Ltd., Hangzhou, China) intramuscularly, followed by drinking 800–1,000 ml of water to dilate the stomach wall before MRI.

All examinations were performed with a whole-body 3.0-T MR scanner (Discovery MR750; GE Healthcare, Milwaukee, WI, USA) equipped with an 8-channel, phased-array body coil. The conventional MRI protocols used for standardized gastric imaging at our institution include the following sequence: 1) axial three-dimensional (3D) spoiled-gradient recalled-echo sequences for liver acquisition with volume acceleration flexible (LAVA-Flex) sequence in one breath-hold; 2) respiratory-triggered axial PROPELLER T2-weighted imaging (T2WI) with fat suppression; 3) axial, coronal, and sagittal single-shot fast spin-echo T2WI in breath-hold; and 4) respiratory-triggered axial DWI sequence included two *b* values (*b* = 0 and 800 s/mm^2^).

IVIM-DWI was performed by using a respiratory-triggered single-shot echo-planar imaging sequence in the transverse plane with diffusion in three orthogonal directions, and the parallel imaging using the array spatial-sensitivity encoding technique (ASSET) was used to shorten the scanning time and reduce image distortion. Ten *b* values from 0 to 1200 s/mm^2^ (0, 10, 20, 40, 100, 200, 400, 800, 1,000, and 1,200) were applied. Quantitative DCE-MRI was performed by using multiphase axial 3D spoiled-gradient recalled-echo sequences for liver acquisition with volume acceleration-extended volume (LAVA-XV) sequence with breath-hold. According to our previous study (19), pre-contrast T1 mapping with four different flip angles (3°, 6°, 9°, and 12°) was acquired before dynamic scanning for the determination of pre-contrast T1 values. Then a dynamic scan with 42 consecutive phases was performed, which shared the scanning parameters and range as T1 mapping, with a flip angle of 15° and temporal resolution of 6 s/phase. A bolus of gadopentetate dimeglumine (Magnevist, Bayer Schering, Berlin, Germany) at a constant dose of 0.1 mmol/kg was power injected, followed by a 20-ml saline flush at a rate of 2.5 ml/s for all patients. The acquisition time was 18 s for each of the three consecutive phases with an interval of 5–10 s; the total scanning time for DCE-MRI was 5–6 min. The detailed acquisition parameters of sequences are shown in **Supplementary Table 1**.

### Imaging Analysis

Two radiologists (YJZ and YL, with 9 and 17 years of experience in gastrointestinal abdominal imaging, respectively) who were blinded to the patients’ clinical and histopathological data independently reviewed the MR imaging and measured the DCE-MRI and IVIM-DWI parameters at the largest section of the tumor with good image quality. The mean values of quantitative parameters were used for subsequent analysis, and the interobserver agreement was also assessed according to the intraclass correlation coefficient (ICC). In order to ensure data repeatability, all parameters were measured twice with a month interval to assess the intraobserver agreement.

The region of interest (ROI) drawing principles were as follows: the slide containing the largest tumor diameter was selected for further analysis. The ROI was manually traced slightly along the borders of the tumor to include the entire tumor, while avoiding visible blood vessels, necrotic areas, and cystic areas, on DCE-MRI and IVIM-DWI.

The IVIM images were transferred to GE ADW 4.6 workstation and analyzed by MADC software in the FuncTool software package. ROI of the tumor was manually delineated on the IVIM-DWI with a *b* value of 800 s/mm^2^ using axial T2WI as a reference. ADC_standard_ value was calculated by the monoexponential model using the total available *b* values according to the following equation:


$${\text{S}}_{b}/{\text{S}}_{0}=\text{ exp}\left(-b\cdot {\text{ADC}}_{\text{standard}}\right)$$

The IVIM parameters were calculated by biexponential fitting according to the following equation, suggested by Le Bihan et al. (13):


$${\text{S}}_{b}/{\text{S}}_{0}=fexp\left(-b\cdot {\text{D}}^{*}\right)+\left(1\;-f\right)exp\left(-b\cdot \text{D}\right)$$

where S_b_ is the signal intensity with diffusion gradient *b* and S_0_ is the signal intensity without diffusion gradient. D is the true diffusion coefficient as reflected by pure water molecular diffusion, D^*^ is the pseudo-diffusion coefficient representing perfusion-related incoherent microcirculation, and *f* is the perfusion fraction related to the microvascular volume fraction. The parameter maps of IVIM were generated automatically by the MADC software, and the ADC_standard_, D, D^*^, and *f* values in the ROIs were obtained

Quantitative DCE-MRI parameters were calculated using an in-house-developed image-processing workstation, OmniKinetics 2.0.10 (GE Healthcare, Beijing, China). The signal intensity on MRI was converted into an equivalent concentration of contrast material using the variable flip angles method. The pharmacokinetic parameters including volume transfer constant (K^trans^), reverse reflux rate constant (k_ep_), extracellular extravascular volume fraction (v_e_), and plasma volume fraction (v_p_), which were derived from DCE-MRI, were calculated using the two-compartment extended Tofts model as described in our previous study and report (19, 20).

### Clinical Treatment

All 129 patients were treated with 4 to 6 cycles of oxaliplatin-based NCT as recommended in CSCO guideline (7), in which 76 patients receiving oxaliplatin and S-1 (SOX) regimen, 32 patients receiving capecitabine and oxaliplatin (XELOX) regimen, and 21 patients receiving docetaxel oxaliplatin and S-1 (DOS) regimen. D2 radical gastrectomy was performed within 30 days after the completion of NCT. The surgical procedures were in accordance with CSCO guidelines for gastric cancer (7). Adjuvant chemotherapy was routinely started 3–4 weeks after surgery, and the oncologist decided on both regimens and cycles based on the clinical and pathological responses.

### Histopathological Examination and Tumor Regression Grade Evaluation

Patient records and original histopathological slides were independently re-evaluated by 2 pathologists with over 10 years’ experience in gastrointestinal pathology. The pathologists were blinded to the routine diagnoses and patient outcomes. Response to chemotherapy was assessed according to the Mandard TRG system (21), which divided the residual tumor into grades 1–5, based on the amount of fibrosis and/or necrosis over the remaining viable tumor cells. To ensure consistency of the evaluation criteria, the 2 pathologists were trained prior to the evaluation. In case of disagreement, a consensus diagnosis would be reached through joint re-review and discussion on a multi-headed microscope. Patients were divided into two groups: pathological responders (pR) (TRG 1–3) and pathological non-responders (pNR) (TRG 4 and 5).

Histopathological variables were also recorded, including histopathological type, tumor differentiation, Lauren classification, lymphovascular invasion (LVI), perineural invasion (PNI), and immunohistochemical assays of HER2, EGFR, and c-MET. For patients with no residual remaining tumor after NCT, a preoperative biopsy specimen was used for analysis. TNM stage was assessed according to the 8th edition of the AJCC staging system. HER2, EGFR, and c-MET expression were detected according to the HER2 detection guideline from the College of American Pathologists (22) and a previous study (23).

### Follow-Up

After radical gastrectomy, all patients were followed up every 3 months for the first year and every 6–12 months afterward. Follow-up consisted of physical examination, tumor marker assessment, CT scan, and endoscopic examination. Recurrence-free survival (RFS) was recorded and was defined as the interval between the date of surgery and the first date of identified local and/or distant recurrence or the last follow-up date without recurrence. Tumor recurrence was defined as local recurrence, distant metastasis, or death caused by gastric cancer, detected by imaging or pathology. All patients were observed until recurrence or the final follow-up date of December 31, 2020. Patients were censored if they were recurrence-free and alive at the last follow-up.

### Statistical Analysis

All statistical analyses were performed with SPSS (version 21.0; SPSS Inc., Chicago, IL, USA). The intraobserver and interobserver reliability in the measurements of IVIM-DWI and DCE-MRI parameters was estimated with ICC, which was defined in previous studies (24).

Quantitative data were expressed as the median and interquartile range (IQR) and were compared using the Mann–Whitney U-test. Categorical data were expressed as number (percentage) and were compared using the *χ*^2^ test or Fisher’s exact test, as appropriate. Differences in clinicopathological features, IVIM-DWI, and quantitative DCE-MRI parameters between the pR and pNR were compared. Those variables with a significant difference, as determined by the univariate logistic regression analysis, were chosen for multivariate logistic regression analysis to identify significant independent predictive parameters for NCT response.

Multivariate logistic regression was used to build a combined prediction model with the statistically significant parameters. The prediction performance of the quantitative parameters and models was evaluated by receiver operating characteristic (ROC) curve, quantified by the area under the curve (AUC), overall accuracy (ACC), sensitivity, specificity, negative predictive value (NPV), and positive predictive value (PPV). The optimal cutoff value was calculated at the maximum value of Youden’s index (sensitivity + specificity − 1).

The quantitative parameters and prediction probabilities were converted into binary variables according to the diagnostic threshold, that is, the predicted treatment response group and non-response group. The RFS was assessed using the Kaplan–Meier method, and differences between predicted groups were assessed by log-rank test. Univariable and multivariable Cox proportional hazards analyses were used to determine independent prognostic factors for tumor recurrence among clinicopathological factors, quantitative parameters, and prediction models. *p* < 0.05 was considered to be statistically significant.

## Results

### The Clinicopathological Findings

**Figure 2:**
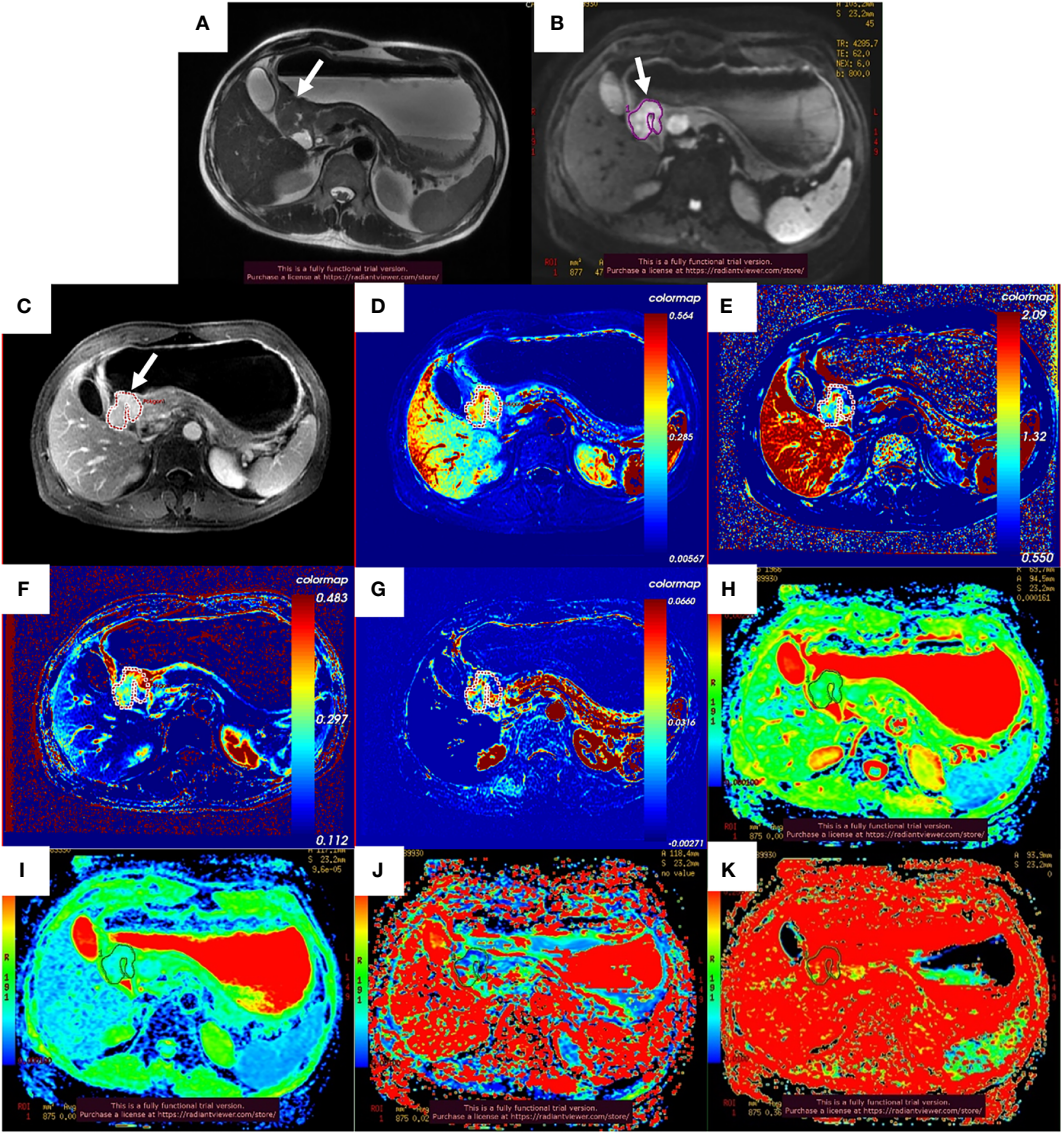
Images of a 51-year-old man with gastric adenocarcinoma in the antrum of the stomach who had response to NCT. In T2WI **(A)**, IVIM-DWI **(B)**, and contrast-enhanced imaging **(C)**, the gastric wall was irregularly thickened with high signal intensity, diffusion restricted, and heterogeneously enhanced (white arrows). The pseudo-colorized K^trans^ maps **(D)**, k_ep_ map **(E)**, v_e_ map **(F)**, v_p_ map **(G)**, ADC_standard_ map **(H)**, D map **(I)**, D^*^ map **(J)**, and *f* map **(K)** show mixed red, green, and blue colors in the corresponding tumor with a K^trans^ of 0.298 min^−1^, k_ep_ of 1.086 min^−1^, v_e_ of 0.303, v_p_ of 0.027, ADC_standard_ of 1.330 × 10^−3^ mm^2^/s, D of 0.893 × 10^−3^ mm^2^/s, D^*^ of 24.000 × 10^−3^ mm^2^/s, and *f* of 36.6%. NCT, neoadjuvant chemotherapy; T2WI, T2-weighted imaging; IVIM-DWI, intravoxel incoherent motion diffusion-weighted imaging.

**Figure 3:**
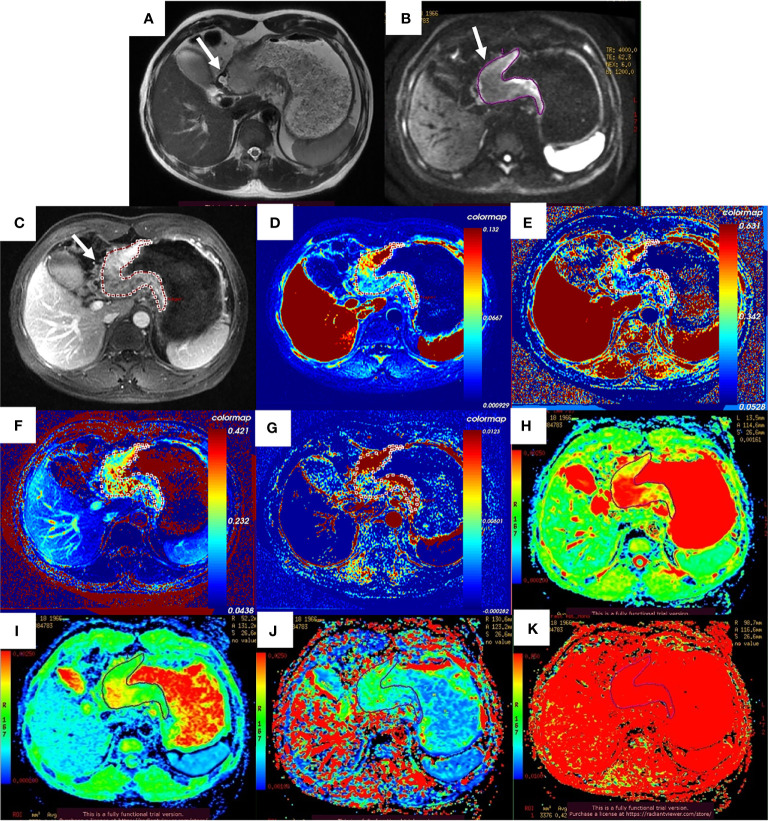
Images of a 52-year-old man with gastric adenocarcinoma in the body of the stomach who had non-response to NCT. In T2WI **(A)**, IVIM-DWI **(B)**, and contrast-enhanced imaging **(C)**, the gastric wall was irregularly thickened with high signal intensity, diffusion restricted, and heterogeneously enhanced (white arrows). The pseudo-colorized K^trans^ maps **(D)**, k_ep_ map **(E)**, v_e_ map **(F)**, v_p_ map **(G)**, ADC_standard_ map **(H)**, D map **(I)**, D^*^ map **(J)**, and *f* map **(K)** show mixed red, green, and blue colors in the corresponding tumor with a K^trans^ of 0.072 min^−1^, k_ep_ of 0.335 min^−1^, v_e_ of 0.271, v_p_ of 0.015, ADC_standard_ of 2.140 × 10^−3^ mm^2^/s, D of 1.500 × 10^−3^ mm^2^/s, D^*^ of 10.700 × 10^−3^ mm^2^/s, and *f* of 42.2%. NCT, neoadjuvant chemotherapy; T2WI, T2-weighted imaging; IVIM-DWI, intravoxel incoherent motion diffusion-weighted imaging.

After the NCT and radical gastrectomy, the complete histopathologic regression of the LAGC (TRG 1) was achieved in 12/129 patients (9.3%); TRG 2 was recorded in 12/129 patients (9.3%), TRG 3 in 45/129 patients (34.9%), TRG 4 in 43/129 patients (33.3%), and TRG 5 in 17/129 (13.2%). According to TRG results, patients were divided into the pR group (n = 69) (**Figure 2**) and the pNR group (n = 60) (**Figure 3**).

There were significant differences in LVI, PNI, and postoperative pathological stage (ypStage) between the pR and pNR groups (all *p* < 0.001). No significant differences were found for gender, age, MTD, tumor site, surgical approach, histopathological type, differentiation, Lauren’s classification, HER2 expression, EGFR expression, and C-MET expression between the two groups (all *p* > 0.05) (**Table 1**).

**Table 1:** Clinical and histopathological characteristics of 129 patients in pR and pNR groups.

Characteristic	All patients (n = 129)	pR (n = 69)	pNR (n = 60)	*p*-Value
Gender				0.407^*^
Male	107 (82.9)	59 (85.5)	48 (80.0)	
Female	22 (17.1)	10 (14.5)	12 (20.0)	
Age, years	60.00 (52.00, 64.50)	60.00 (52.00, 64.00)	61.00 (48.25, 67.75)	0.806^**^
MTD, cm	4.80 (3.90, 5.80)	4.70 (3.75, 5.95)	5.00 (3.93, 5.58)	0.923^**^
Location				0.804^*^
EGJ	43 (33.3)	22 (31.9)	21 (35.0)	
Fundus	7 (5.4)	5 (7.3)	1 (1.7)	
Body	27 (20.9)	13 (18.8)	14 (23.3)	
Antrum	35 (27.1)	21 (30.4)	14 (23.3)	
Whole stomach	17 (13.3)	8 (11.6)	10 (16.7)	
Surgical approach				0.834^*^
Esophagogastrectomy	29 (22.5)	15 (21.7)	14 (23.3)	
Proximal gastrectomy	14 (10.9)	7 (10.1)	7 (11.7)	
Distal gastrectomy	44 (34.1)	26 (37.7)	18 (30.0)	
Total gastrectomy	42 (32.6)	21 (30.4)	21 (35.0)	
Histopathological type				0.576^***^
Adenocarcinoma	104 (80.6)	58 (84.1)	46 (76.7)	
Mucinous	7 (5.4)	3 (4.3)	4 (6.7)	
Signet-ring cell	18 (14.0)	8 (11.6)	10 (16.6)	
Differentiation				0.937^***^
Well	3 (2.3)	2 (2.9)	1 (1.7)	
Moderate	36 (27.9)	20 (29.0)	16 (26.7)	
Poor	90 (69.8)	47 (68.1)	43 (71.6)	
Lauren classification				0.165^*^
Intestinal	51 (39.5)	27 (39.1)	24 (40.0)	
Diffuse	43 (33.3)	19 (27.5)	24 (40.0)	
Mixed	35 (27.2)	23 (33.4)	12 (20.0)	
Lymphovascular invasion				<0.001^*^
Positive	59 (45.7)	18 (26.1)	41 (68.3)	
Negative	70 (54.3)	51 (73.9)	19 (31.7)	
Perineural invasion				<0.001^*^
Positive	72 (55.8)	25 (36.2)	47 (78.3)	
Negative	57 (44.2)	44 (63.8)	13 (21.7)	
HER2 expression				0.514^***^
−/1+	101 (78.3)	53 (76.8)	48 (80.0)	
2+	20 (15.5)	10 (14.5)	10 (16.7)	
3+	8 (6.2)	6 (8.7)	2 (3.3)	
EGFR expression				0.423^*^
−/1+	81 (62.8)	46 (66.7)	35 (58.3)	
2+	37 (28.7)	19 (27.5)	18 (30.0)	
3+	11 (8.5)	4 (5.8)	7 (11.7)	
c-MET expression				0.881^***^
−/1+	92 (71.3)	48 (69.6)	44 (73.3)	
2+	33 (25.6)	19 (27.5)	14 (23.4)	
3+	4 (3.1)	2 (2.9)	2 (3.3)	
ypStage				<0.001^*^
0/I	33 (25.6)	30 (43.5)	3 (5.0)	
II	43 (33.3)	24 (34.8)	19 (31.7)	
III	53 (41.1)	15 (21.7)	38 (63.3)	

Data are given as n (%) or median (IQR).

pR, pathological responders; pNR, pathological non-responders; MTD, maximum tumor diameter; EGJ, esophagogastric junction.

^*^p-Values were calculated using χ^2^ test.

^**^p-Values were calculated using Mann–Whitney U-test.

^***^p-Values were calculated using Fisher’s exact test.

### Intraobserver and Interobserver Agreement Assessments for Quantitative Analysis

The interobserver and intraobserver agreement for the assessments of quantitative parameters by the two radiologists is shown in **Supplementary Table 2**. ICCs for interobserver and intraobserver were all above 0.80 (95% CI, 0.837–0.975, and 0.845–0.966, respectively), which indicated excellent agreement. Therefore, the mean values of the first measurement by the two radiologists were used for subsequent analysis.

### Comparison of Dynamic Contrast-Enhanced MRI and Intravoxel Incoherent Motion Diffusion-Weighted Imaging Quantitative Parameters Between Pathological Response and Pathological Non-Response Groups

**Figure 4:**
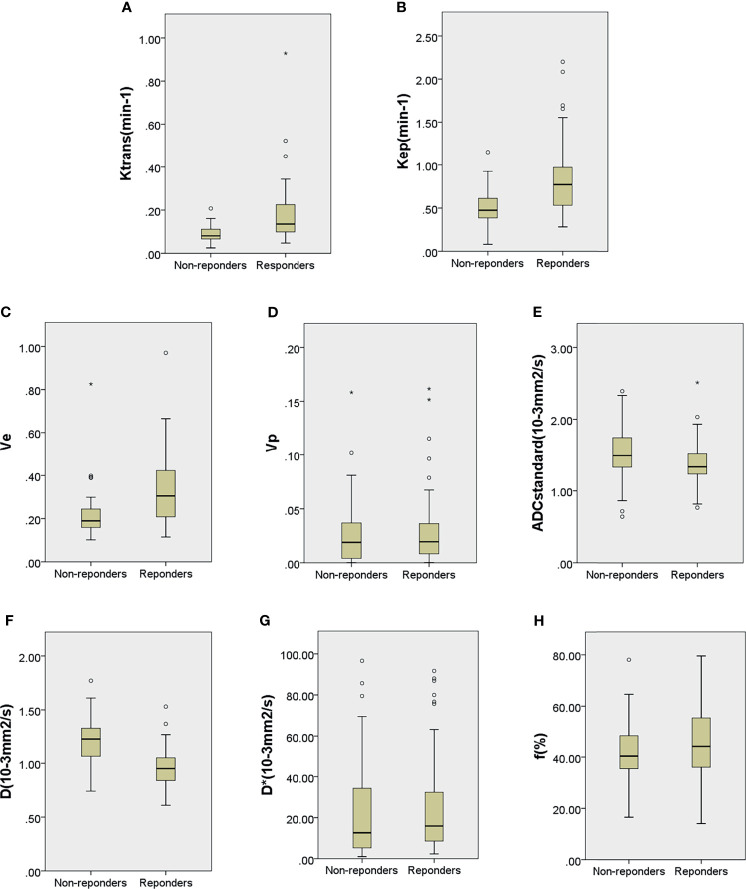
Boxplots for K^trans^ **(A)**, k_ep_ **(B)**, v_e_ **(C)**, v_p_ **(D)**, ADC_standard_ **(E)**, D **(F)**, D^*^ **(G)**, and *f* **(H)** of locally advanced gastric cancer in the pathological responder and non-responder groups. The top and bottom of the boxes are the 25th and 75th percentiles, respectively. The mid lines and bars indicate the medians and the 5th–95th percentiles, respectively. Circles indicate outliers, stars represent extreme values.

Among the 129 patients, quantitative DCE-MRI parameters in the primary lesion of K^trans^, k_ep_, v_e_, and v_p_ were 0.103 (0.073, 0.161) min^−1^, 0.592 (0.452, 0.813) min^−1^, 0.229 (0.170, 0.351), and 0.019 (0.007, 0.037); IVIM parameters of ADC_standard_, D, D^*^, and *f* value were 1.380 (1.250, 1.650) × 10^−3^ mm^2^/s, 1.050 (0.920, 1.255) × 10^−3^ mm^2^/s, 15.400 (6.405, 34.300) × 10^−3^ mm^2^/s, and 41.700% (35.550%, 53.550%). The comparisons of quantitative DCE-MRI and IVIM-DWI parameters between the pR and pNR groups are summarized in **Table 2**. The results showed that K^trans^, k_ep_, and v_e_ values in the pR group were significantly higher than those in the pNR group (all *p* < 0.001), while ADC_standard_ and D values were significantly lower than those in the pNR group (*p* = 0.011 and *p* < 0.001). D^*^ value and *f* value in the pR group were slightly higher than those in pNR, but there were no significant differences (*p*-value was 0.233 and 0.105, respectively). v_p_ value showed no significant difference between the two groups (*p* = 0.470). Box and whisker plot graphs for parameters derived from DCE-MRI and IVIM-DWI in the pR and pNR groups are given in **Figure 4**.

**Table 2:** Comparison of DCE-MRI and IVIM quantitative parameters between pR and pNR groups.

Parameter	pR (n = 69)	pNR (n = 60)	Z	*p*-Value
K^trans^ (min^−1^)	0.135 (0.097, 0.226)	0.081 (0.066, 0.111)	−5.227	<0.001^*^
k_ep_ (min^−1^)	0.772 (0.532, 0.987)	0.475 (0.383, 0.614)	−5.241	<0.001^*^
v_e_	0.305 (0.205, 0.428)	0.189 (0.158, 0.248)	−4.835	<0.001^*^
v_p_	0.019 (0.008, 0.037)	0.019 (0.004, 0.038)	−0.722	0.470^*^
ADC_standard_ (10^−3^ mm^2^/s)	1.340 (1.230, 1.525)	1.495 (1.333, 1.745)	−2.541	0.011^*^
D (10^−3^ mm^2^/s)	0.950 (0.845, 1.065)	1.230 (1.058, 1.330)	7.330	<0.001^*^
D^*^ (10^−3^ mm^2^/s)	15.900 (8.200, 34.800)	12.650 (5.240, 34.900)	−1.192	0.233^*^
*f* (%)	44.200 (35.650, 55.550)	40.450 (35.475, 48.575)	−1.724	0.105^*^

Data were expressed as the median (IQR).

K^trans^, volume transfer constant; k_ep_, reflux rate; v_e_, volume fraction of the extravascular extracellular matrix; v_p_, plasma volume fraction; ADC_standard_, standard apparent diffusion coefficient; D, true diffusion coefficient; D^*^, pseudo-diffusion coefficient; f, microvascular volume fraction; DCE-MRI, dynamic contrast-enhanced MRI; IVIM, intravoxel incoherent motion; pR, pathological responders; pNR, pathological non-responders.

^*^p-Values were calculated using the Mann–Whitney U-test.

### Clinical Factors and MRI Quantitative Parameters for Predicting Neoadjuvant Chemotherapy Response

With the univariate analysis, none of the clinical factors showed significant correlation with pathology response of NCT (all *p* > 0.05). Meanwhile, higher pretreatment baseline K^trans^ (OR = 9.334; 95% CI, 3.531–24.672), k_ep_ (OR = 4.442; 95% CI, 2.297–8.589), and v_e_ values (OR = 3.221; 95% CI, 1.837–5.646) and lower ADC_standard_ (OR = 0.673; 95% CI, 0.465–0.973) and D values (OR = 0.221; 95% CI, 0.127–0.384) were more likely to be responsive to NCT (all *p* < 0.05).

Multivariate logistic regression analysis demonstrated that K^trans^ (OR = 5.300; 95% CI, 1.470–19.104), k_ep_ (OR = 3.918; 95% CI, 1.484–10.345), v_e_ values (OR = 2.926; 95% CI, 1.437–5.961), and D values (OR = 0.266; 95% CI, 0.138–0.515) were independently associated with the response to NCT. The univariate and multivariate logistic regression results are summarized in **Supplementary Table 3**.

### Prediction Efficiency of Response to Neoadjuvant Chemotherapy Using Dynamic Contrast-Enhanced MRI and Intravoxel Incoherent Motion Diffusion-Weighted Imaging Quantitative Parameters

Multivariate logistic regression analysis was conducted to build prediction models for response to NCT, using K^trans^, k_ep_, and v_e_ for DCE-MRI model; D for the IVIM-DWI model; and K^trans^, k_ep_, v_e_, and D for the DCE+IVIM model. The results are summarized in **Table 3**.

**Table 3:** Model of DCE-MRI and IVIM-DWI quantitative parameters and their combination in predictive impact on response to NCT by multivariate logistic regression analysis.

Model^*^	Coefficients	Std. error	Wald	OR	95% CI	*p*-Value
DCE-MRI						
K^trans^	1.744	0.587	8.816	5.722	1.809–18.096	0.003
k_ep_	1.258	0.413	9.272	3.517	1.565–7.902	0.002
v_e_	1.257	0.359	12.240	3.516	1.738–7.111	<0.001
Constant	0.852	0.306	7.727	2.344		0.005
IVIM-DWI						
D	−1.509	0.281	28.760	0.221	0.127–0.384	<0.001
Constant	0.156	0.212	0.538	0.463	1.168	
Combined						
K^trans^	1.668	0.654	6.499	5.300	1.470, 19.104	0.011
k_ep_	1.365	0.495	7.596	3.918	1,484, 10.345	0.006
v_e_	1.074	0.363	8.752	2.926	1.437, 5.961	0.003
D	−1.323	0.336	15.472	0.266	0.138, 0.515	<0.001
Constant	0.966	0.353	7.476	2.627		0.006

OR, odds ratio; K^trans^, volume transfer constant; k_ep_, reflux rate; v_e_, volume fraction of the extravascular extracellular matrix; D, true diffusion coefficient; DCE-MRI, dynamic contrast-enhanced MRI; IVIM-DWI, intravoxel incoherent motion diffusion-weighted imaging; NCT, neoadjuvant chemotherapy.

^*^Forward stepwise, likelihood ratio method was adapted in multivariate logistic regression analysis, with probability <0.05 for stepwise entry and 0.1 for removal.

**Figure 5:**
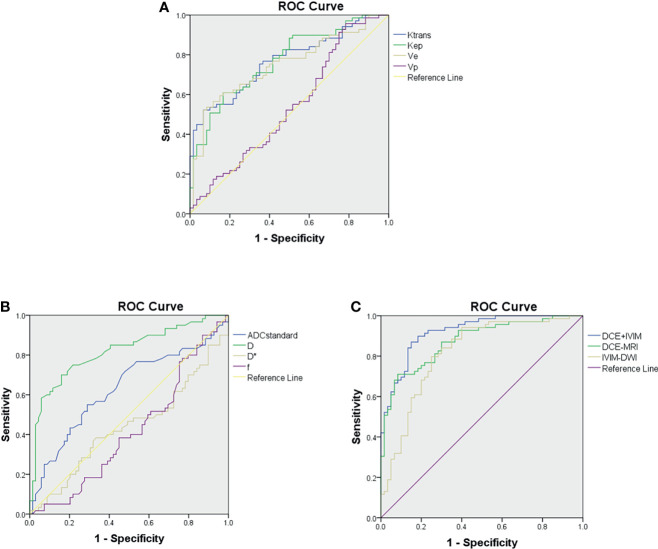
Receiver operating characteristic curves for predicting treatment response to neoadjuvant therapy. **(A)** Comparison of diagnostic performance using dynamic contrast-enhanced (DCE) MRI parameters. **(B)** Comparison of diagnostic performance using intravoxel incoherent motion (IVIM) parameters. **(C)** Comparison of diagnostic performance using combined model of DCE-MRI and IVIM-DWI.

ROC curve analysis results of MRI quantitative parameters and combined model for predicting pathological treatment response are presented in **Table 4** and **Figure 5**. D value was the single parameter with the highest predictive efficiency, of which AUC was 0.812. The combination of DCE-MRI and IVIM-DWI displayed the highest AUC of 0.922.

**Table 4:** Diagnostic performance of DCE-MRI and IVIM-DWI quantitative parameters and their combinations in discriminating treatment response to NCT in LAGC patients.

Parameters or model	Cutoff value	AUC	Sensitivity	Specificity	Accuracy	PPV	NPV	*p*
K^trans^ (min^−1^)	0.134	0.767 (0.687–0.848)	52.2	93.3	71.3	90.0	62.9	<0.001
k_ep_ (min^−1^)	0.661	0.768 (0.688–0.848)	60.9	83.3	71.3	80.8	64.9	<0.001
v_e_	0.300	0.747 (0.662–0.832)	52.2	93.3	71.3	90.0	62.9	<0.001
v_p_	0.002	0.537 (0.436–0.638)	95.7	21.7	61.2	58.4	81.3	0.470
ADC_standard_ (10^−3^ mm^2^/s)	1.440	0.630 (0.531–0.728)	71.0	55.0	55.0	64.5	62.3	0.011
D (10^−3^ mm^2^/s)	1.200	0.812 (0.736–0.889)	92.8	60.0	77.5	72.7	87.8	<0.001
D^*^ (10^−3^ mm^2^/s)	10.650	0.561 (0.460–0.662)	72.5	46.7	60.5	63.6	59.6	0.233
*f* (%)	50.900	0.583 (0.485–0.681)	36.2	81.7	57.4	69.4	52.7	0.105
DCE^*^	0.613	0.875 (0.816–0.934)	71.0	91.7	80.6	90.7	73.3	<0.001
IVIM^*^	0.462	0.818 (0.743–0.894)	84.1	70.0	77.5	76.3	79.2	<0.001
DCE+IVIM^*^	0.481	0.922 (0.877–0.966)	87.0	85.0	86.0	87.0	85.0	<0.001

Data in parentheses are 95% CI.

AUC, area under the curve; PPV, positive predictive value; NPV, negative predictive value; K^trans^, volume transfer constant; k_ep_, reflux rate; v_e_, volume fraction of the extravascular extracellular matrix; v_p_, plasma volume fraction; ADC_standard_, standard apparent diffusion coefficient; D, true diffusion coefficient; D^*^, pseudo-diffusion coefficient; f, microvascular volume fraction; DCE-MRI, dynamic contrast-enhanced MRI; IVIM-DWI, intravoxel incoherent motion diffusion-weighted imaging; NCT, neoadjuvant chemotherapy; LAGC, locally advanced gastric cancer.

^*^Cutpoint is probability.

### Prognostic Value of MRI Quantitative Parameters and Its Association With Recurrence-Free Survival

The median follow-up period for all patients was 15.0 months (IQR, 9.0–21.0 months). Of the 129 patients, 54 patients (41.9%) developed tumor recurrence by the last follow-up day. The median RFS time was 24.0 months (95% CI: 17.2–30.8), and the 2-year RFS rate was 49.9% months.

Univariate Cox proportional hazards analysis with clinicopathological factors, MRI quantitative parameters, and prediction models showed that signet ring cell, LVI, PNI, ypStage III, pathological response, K^trans^ value, k_ep_ value, v_e_ value, D value, DCE model, IVIM model, and DCE+IVIM model were significantly associated with RFS. Multivariate Cox regression analysis showed that ypStage III (hazard ratio [HR] = 6.197; 95% CI, 2.132–18.014) and no response predicted by IVIM model (HR = 2.240; 95% CI, 1.231–4.075) were independent predictors of poor RFS (**Table 5**).

**Table 5:** Univariate and multivariate Cox proportional hazards prediction analyses of recurrence-free survival according to responder and non-responder groups determined by baseline DCE-MRI and IVIM-DWI quantitative parameters and their combination models.

Parameters	Univariate analysis			Multivariate analysis		
	Hazard ratio	95% CI	*p*	Hazard ratio	95% CI	*p*
Clinical factors						
Gender						
Male	1 (reference)					
Female	1.436	0.755–2.733	0.270			
Age, years	0.982	0.959–1.005	0.121			
MTD, cm	1.125	0.941–1.345	0.196			
Location						
EGJ	1 (reference)					
Fundus	2.536	0.843–7.624	0.098			
Body	1.161	0.553–2.434	0.694			
Antrum	0.728	0.336–1.579	0.421			
Whole stomach	1.571	0.712–3.467	0.264			
Pathological factors						
Histopathological type						
Adenocarcinoma	1 (reference)					
Mucinous	0.900	0.217–3.745	0.885			NS
Signet-ring cell	2.396	1.227–4.680	0.010^**^			NS
Differentiation						
Well	1 (reference)					
Moderate	1.447	0.190–10.989	0.721			
Poor	1.529	0.209–11.157	0.676			
Lauren classification						
Intestinal	1 (reference)					
Diffuse	1.398	0.767–2.549	0.274			
Mixed	0.704	0.337–1.471	0.350			
Lymphovascular invasion						
Negative	1 (reference)					
Positive	3.051	1.738–5.356	<0.001^**^			NS
Perineural invasion						
Negative	1 (reference)					
Positive	2.639	1.435–4.854	0.002^**^			NS
HER2 expression						
−/1+	1 (reference)					
2+	0.877	0.411–1.869	0.733			
3+	1.094	0.392–3.054	0.863			
EGFR expression						
−/1+	1 (reference)					
2+	0.617	0.321–1.185	0.147			
3+	0.965	0.379–2.460	0.941			
c-MET expression						
−/1+	1 (reference)					
2+	0.570	0.286–1.138	0.111			
3+	1.621	0.390–6.736	0.506			
ypStage						
0/I	1 (reference)			1 (reference)		
II	2.220	0.715–6.892	0.168	1.930	0.618-6.026	0.257
III	8.834	3.145–24.812	<0.001^**^	6.197	2.132-18.014	0.001
Pathological response						
Responder	1 (reference)					
Non-responder	2.887	1.637–5.092	<0.001^**^			NS
Multiparametric MRI						
K^trans^ (min^−1^)						
Responder (>0.134)	1 (reference)					
Non-responder (≤0.134)	3.125	1.473–6.631	0.003^**^			NS
k_ep_ (min^−1^)						
Responder (>0.661)	1 (reference)					
Non-responder (≤0.661)	2.624	1.381–4.986	0.003^**^			NS
v_e_						
Responder (>0.300)	1 (reference)					NS
Non-responder (≤0.300)	2.369	1.191–4.714	0.014^**^			
v_p_						
Responder (>0.002)	1 (reference)					
Non-responder (≤0.002)	1.442	0.704–2.955	0.317			
ADC_standard_ (×10^−3^ mm^2^/s)						
Responder (<1.440)	1 (reference)					
Non-responder (≥1.440)	1.593	0.933–2.720	0.088			
D (×10^−3^ mm^2^/s)						
Responder (<1.200)	1 (reference)					
Non-responder (≥1.200)	3.746	2.171–6.462	<0.001^**^			NS
D^*^ (×10^−3^ mm^2^/s)						
Responder (>10.650)	1 (reference)					
Non-responder (≤10.650)	1.434	0.838–2.456	0.188			
*f* (%)						
Responder (>50.900)	1 (reference)					
Non-responder (≤50.900)	1.266	0.678–2.364	0.459			
DCE^*^						
Responder (>0.613)	1 (reference)					
Non-responder (≤0.613)	2.522	1.351–4.707	0.004^**^			NS
IVIM^*^						
Responder (>0.462)	1 (reference)			1 (reference)		
Non-responder (≤0.462)	3.646	2.065–6.438	<0.001^**^	2.240	1.231-4.075	0.008
DCE+IVIM^*^						
Responder (>0.481)	1 (reference)					
Non-responder (≤0.481)	2.789	1.582–4.916	<0.001^**^			NS

NS, not significant; K^trans^, volume transfer constant; k_ep_, reflux rate; v_e_, volume fraction of the extravascular extracellular matrix; v_p_, plasma volume fraction; ADC_standard_, standard apparent diffusion coefficient; D, true diffusion coefficient; D^*^, pseudo-diffusion coefficient; f, microvascular volume fraction; DCE-MRI, dynamic contrast-enhanced MRI; IVIM-DWI, intravoxel incoherent motion diffusion-weighted imaging; MTD, maximum tumor diameter; EGJ, esophagogastric junction.

^*^The predictive probability of the combined model was used.

^**^Data are statistically from the univariate Cox proportional hazards analyses.

**Figure 6:**
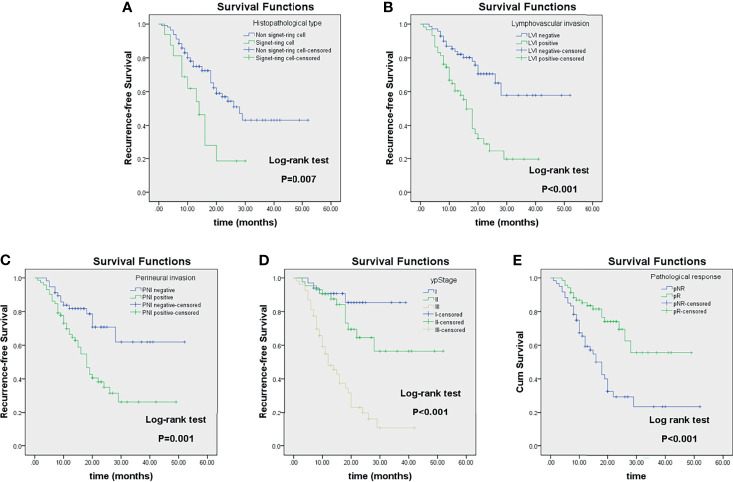
Kaplan–Meier analyses of recurrence-free survival (RFS) in locally advanced gastric cancer (LAGC) patients (n = 129) stratified by category: histopathological type **(A)**, lymphovascular invasion **(B)**, perineural invasion **(C)**, ypStage **(D)**, and pathological response **(E)**.

**Figure 7:**
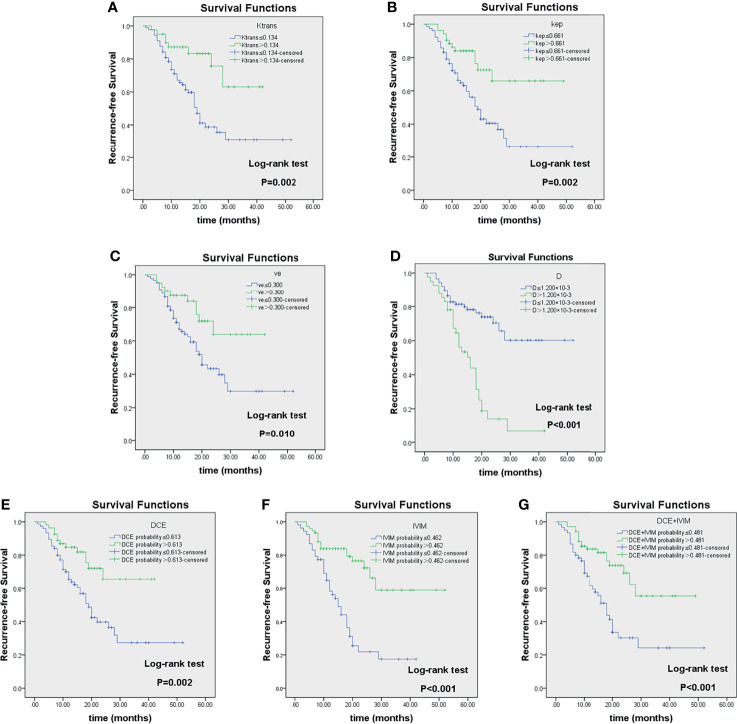
Kaplan–Meier analyses of recurrence-free survival (RFS) in LAGC patients (n = 129) stratified by category: responders and non-responders as classified according to imaging parameter cutoff values of K^trans^ **(A)**, k_ep_ **(B)**, v_e_ **(C)**, D **(D)**, DCE-MRI **(E)**, IVIM-DWI **(F)**, and their combinations **(G)**. Reported in **Table 4**. LAGC, locally advanced gastric cancer; DCE-MRI, dynamic contrast-enhanced MRI; IVIM-DWI, intravoxel incoherent motion diffusion-weighted imaging.

The Kaplan–Meier survival analysis based on clinicopathological factors, MRI quantitative parameters, and prediction model, which were identified from Cox regression analysis, are summarized in **Tables 6**, **7**. These features significantly stratified in 2-year RFS rate are demonstrated in **Figures 6**, **7** according to the log-rank test (all log-rank *p* < 0.05).

**Table 6:** Kaplan–Meier survival analysis according to pathological factors for predictors of RFS.

Pathological factors	N = 129	Median RFS (95% CI)	2-year RFS rate	Log-rank *p*
Histopathological type				0.007
Signet-ring cell	16	14 (9.431–18.569)	18.6 ± 11.5	
Non-signet-ring cell	113	28 (21.011–34.989)	54.4 ± 5.9	
Lymphovascular invasion				<0.001
Positive	59	16 (13.501–18.499)	24.5 ± 7.4	
Negative	70	52^*^	70.5 ± 6.3	
Perineural invasion				0.001
Positive	72	18 (14.793–21.207)	35.0 ± 6.8	
Negative	57	52^*^	70.8 ± 7.5	
ypStage				<0.001
0/I	33	39^*^	85.4 ± 7.1	
II	43	52^*^	64.5 ± 9.1	
III	53	12 (7.719–16.281)	19.8 ± 6.6	
Pathological response				<0.001
Responder	69	49^*^	69.4 ± 7.2	
Non-responder	60	16 (12.221–19.779)	29.2 ± 7.0	

RFS, recurrence-free survival.

^*^Cumulative survival probability was above the follow-up.

**Table 7:** Kaplan–Meier survival analysis according to cutoff values for predictors of RFS.

Parameters	Above cutoff			Below cutoff			Log-rank *p*
	Median parameter (25%, 75% quartile)	Median RFS (n)^**^	2-year RFS rate	Median parameter (25%, 75% quartile)	Median RFS (n)	2-year RFS rate	
DCE-MRI parameters							
K^trans^	0.212 (0.163, 0.266)	42^***^ (40)	75.7 ± 9.3	0.086 (0.066, 0.104)	19 (89)	38.8 ± 6.3	0.002
k_ep_	0.869 (0.774, 1.068)	49^***^ (52)	66.0 ± 9.4	0.467 (0.379, 0.573)	19 (77)	40.6 ± 6.5	0.002
v_e_	0.410 (0.361, 0.490)	42^***^ (40)	63.9 ± 10.5	0.189 (0.160, 0.235)	20 (89)	43.4 ± 6.4	0.010
v_p_	0.026 (0.012, 0.400)	28 (113)	53.4 ± 5.9	0.001 (0.000, 0.002)	18 (16)	26.7 ± 13.1	0.308
IVIM-DWI parameters							
ADC_standard_	1.680 (1.550, 1.895)	18 (53)	38.7 ± 7.6	1.270 (1.150, 1.360)	28 (76)	58.6 ± 7.7	0.081
D	1.310 (1.265, 1.395)	16 (41)	14.1 ± 6.5	0.955 (0.850, 1.058)	52^***^ (88)	70.4 ± 6.1	<0.001
D^*^	28.300 (16.200, 52.925)	52^***^ (82)	57.8 ± 6.3	5.300 (3.370, 7.290)	20 (47)	39.3 ± 9.1	0.180
* f*	56.750 (54.725, 63.375)	28 (36)	63.7 ± 9.1	37.500 (32.800, 43.650)	22 (93)	45.6 ± 6.4	0.452
Response prediction model^*^							
DCE	0.941 (0.762, 0.990)	42^***^ (54)	65.5 ± 9.1	0.239 (0.154, 0.449)	19 (75)	39.7 ± 6.6	0.002
IVIM	0.755 (0.615, 0.854)	52^***^ (76)	72.3 ± 6.7	0.224 (0.158, 0.358)	15 (53)	21.8 ± 6.8	<0.001
DCE+IVIM	0.910 (0.722, 0.994)	49^***^ (69)	69.3 ± 7.3	0.140 (0.059, 0.258)	18 (60)	30.2 ± 7.1	<0.001

RFS, recurrence-free survival; K^trans^, volume transfer constant; k_ep_, reflux rate; v_e_, volume fraction of the extravascular extracellular matrix; v_p_, plasma volume fraction; ADC_standard_, standard apparent diffusion coefficient; D, true diffusion coefficient; D^*^, pseudo-diffusion coefficient; f, microvascular volume fraction; DCE-MRI, dynamic contrast-enhanced MRI; IVIM-DWI, intravoxel incoherent motion diffusion-weighted imaging.

^*^The predictive probabilities of combined parameters for NCT response derived from multivariate logistic regression analysis previously were used as new parameters.

^**^Data in parentheses are number of patients.

^***^Cumulative survival probability was above the follow-up.

Further subgroup analysis showed that in ypStage II/III, LVI-positive and PNI-positive groups, and 2-year RFS rate between different groups of pathological treatment response and prediction models were also significantly different (all log-rank *p* < 0.05) (**Table 8** and **Supplementary Figure 1**).

**Table 8:** Subgroup Kaplan–Meier survival analysis of RFS according to response grouped by pathology and prediction models.

Subgroup	R		NR		Log-rank *p*
	Median RFS (n)^*^	2-year RFS rate	Median RFS (n)	2-year RFS rate	
ypStage II/III					
Pathological response	28 (39)	59.8 ± 10.0	16 (57)	25.6 ± 6.9	0.006
DCE	42^**^ (35)	57.5 ± 12.1	16 (61)	29.6 ± 6.7	0.004
IVIM	28 (49)	64.2 ± 8.7	15 (47)	17.1 ± 6.4	<0.001
DCE+IVIM	28 (44)	60.1 ± 9.8	16 (52)	25.0 ± 6.8	0.005
LVI positive					
Pathological response	41^**^ (18)	51.3 ± 16.3	14 (41)	15.9 ± 7.2	0.012
DCE	41^**^ (18)	54.3 ± 17.0	14 (41)	15.5 ± 7.1	0.005
IVIM	41^**^ (26)	53.8 ± 15.2	12 (33)	9.9 ± 6.1	0.002
DCE+IVIM	41^**^ (22)	50.6 ± 15.9	14 (37)	15.5 ± 7.1	0.008
PNI positive					
Pathological response	24 (25)	53.2 ± 12.4	16 (47)	24.5 ± 7.6	0.041
DCE	24 (23)	47.2 ± 14.6	16 (49)	29.8 ± 7.4	0.041
IVIM	26 (29)	60.4 ± 12.1	15 (43)	18.8 ± 7.0	0.008
DCE+IVIM	26 (29)	50.4 ± 11.9	16 (43)	25.4 ± 7.9	0.049

R, responders; NR, non-responders; RFS, recurrence-free survival; DCE, dynamic contrast enhanced; IVIM, intravoxel incoherent motion.

^*^Data in parentheses are number of patients.

^**^Cumulative survival probability was above the follow-up.

## Discussion

In this study, DCE-MRI and IVIM-DWI quantitative parameters were used to construct a prediction model for NCT pathological treatment response of LAGC patients, and the relationship between predicted results and RFS was explored for the first time. The results showed that K^trans^, k_ep_, and v_e_ values of DCE-MRI and D values of IVIM-DWI were independent predictors of pathological response to NCT. The prediction models showed good predictive efficacy for NCT response, and RFS could be stratified based on the prediction result.

CSCO guideline of gastric cancer recommends NCT for patients with resectable GC with clinical-stage ≥ cT3-4N1-3M0 (Evidence 1B) (7), which is an important part of the multidisciplinary management for LAGC. At present, morphology-based Response Evaluation Criteria in Solid Tumors (RECIST) can only evaluate and monitor the treatment response through tumor size changes but cannot predict the efficacy of NCT before treatment.

The pathological staging of gastric cancer is an important factor affecting the prognosis of gastric cancer (25); LVI, PNI, and signet-ring cell carcinoma are also indicators for poor prognosis (26, 27). In this study, it was concluded that higher ypStage, LVI positive, PNI positive, and signet ring cell carcinoma were risk factors for poor prognosis of RFS. However, multivariate Cox regression analysis showed that only ypStage and the IVIM-DWI model were independent predictors for RFS, possibly because tumor stage and the IVIM-DWI model had stronger effects on prognosis.

Quantitative DCE-MRI reflects the exchange of contrast agents in tumor blood vessels and extravascular extracellular space through certain pharmacokinetic models (20) and evaluates tumor microvascular structure, capillary permeability, and tissue perfusion. IVIM-DWI uses the biexponential model to quantitatively separate the Brownian motion of water molecules in tissues (diffusion) from the movement of blood in the microvasculature (perfusion) (14), which can reflect the tissue diffusion and microcirculation perfusion more accurately and comprehensively. Studies have shown that perfusion of tumor tissue might be a key factor affecting the sensitivity of some chemotherapy drugs (28, 29). Quantitative DCE-MRI and IVIM-DWI have been widely applied to predict and evaluate the therapeutic response of chemotherapy, radiotherapy, and targeted therapy for a variety of tumors (16, 30, 31). At present, studies on quantitative DCE-MRI and IVIM-DWI mostly focus on head and neck, breast, and pelvic tumors. As gastric MRI is susceptible to artifacts caused by respiratory movement and gastrointestinal peristalsis, the application in the stomach is limited. Studies on NCT response prediction of gastric cancer using quantitative DCE-MRI and IVIM-DWI have not been reported.

The present study showed that the values of K^trans^, k_ep_, and v_e_ in the pR group were significantly higher than in the pNR group before treatment (all *p* < 0.001). K^trans^ reflects the exchangeability of contrast agents in the plasma and extracellular space of tumor tissue, while k_ep_ reflects the flux rate of contrast agent diffusion back into the blood vessels, both of which are important markers of vascular permeability. v_e_ and v_p_ reflect the volume of extravascular extracellular space and plasma in unit voxel, respectively (20). Tong et al. (32) found in a study of rectal cancer that K^trans^, k_ep_, and v_e_ before NCT were significantly higher in the pathological complete response (pCR) group than in the non-pCR group, while these parameters showed no significant difference after treatment. Tang et al. (33) also found that K^trans^ and k_ep_ in the response group were significantly higher than in the non-response group in pancreatic cancer. In addition, K^trans^ and v_e_ were found to be significantly correlated with 3-year progression-free and OS of oropharyngeal and hypopharyngeal squamous cell carcinoma (34). These results are consistent with the findings of our study. High K^trans^ and k_ep_ values reflect higher permeability and perfusion of tumor tissue due to tumor neoangiogenesis, which could make chemotherapy drugs penetrate easier into tumor tissues and kill tumor cells. An increased v_e_ indicates an elevated fraction in the extracellular extravascular space, which might be caused by increasing immature incompetent vessel leakage, which could provide wider distribution space for chemotherapy drugs and more oxygen distribution for tissues to avoid the occurrence of hypoxia, thus increasing the sensitivity of chemotherapy drugs. In a study of glioma, Kim et al. (35) found that v_p_ in the progression group was higher, suggesting that it may be related to the destruction of the blood–brain barrier and tumor angiogenesis. However, our study did not find a significant relationship between NCT treatment response and RFS. We speculate that there might be more influence factors to v_p_, and gastric cancer is not a tumor of rich blood supply. Therefore, the significance of v_p_ in the prediction of NCT treatment response and prognosis of gastric cancer needs to be further clarified.

IVIM-DWI has been widely applied in the diagnosis, treatment response evaluation, and prognosis prediction of various tumors, and its quantitative parameter has been found to be predictive for the prognosis of many cancers (31, 36, 37). In this study, the ADC_standard_ and D values of the pR group were significantly lower than those of the pNR group (both *p* < 0.05), and the multivariate logistic regression revealed that D was an independent predictor for NCT response with an OR of 0.266, indicating that patients with low D values were more likely to respond to NCT. Similar results were also found in the study on response prediction of neoadjuvant therapy for locally advanced rectal cancer (36). Compared with the non-pCR group, the pCR group showed lower pretreatment ADC_mean_, D value, and higher *f* value, and D value was the best predictor of treatment response. The study conducted by Zheng et al. (37) also demonstrated that the residual tumor group had higher ADC value and D value as compared with the non-residual tumor group, and multivariate analysis showed that the pretreatment D value was an independent prognostic factor for cervical cancer. The reason might be that a lower ADC or D value indicated more restriction of water molecule diffusion in tumor tissues, higher cellular density, and richer blood supply. On the contrary, an increased ADC or D value reflects a decrease in cell density of tumor tissue due to necrosis, inflammation, or fibrosis and then affects the penetration and distribution of antitumor drugs as a result of decreased blood supply, ultimately leading to chemotherapy or radiotherapy resistance and poor prognosis. Perfusion-related parameters D^*^ and *f* values have also been found to be possible predictors of tumor treatment response in some studies (37–39). However, in this study, although D^*^ and *f* values in the pR group were higher than those in the pNR group, no significant difference was observed, which may be related to the small sample size or different pathological types of tumors.

The present study combined DCE-MRI and IVIM-DWI quantitative parameters for the first time, to build the prediction model for NCT response in LAGC through multivariate logistic regression. ROC analysis showed that combination DCE-MRI and IVIM-DWI exhibited the highest predictive efficiency, with AUC, sensitivity, and specificity of 0.922, 87.0%, and 85.0%, respectively. To further verify the relationship between MRI quantitative parameters and patient prognosis, Cox regression analysis was performed, and the results showed that the IVIM-DWI model was an independent predictor of RFS. Kaplan–Meier survival analysis showed that low K^trans^, k_ep_, and v_e_ and high D value groups had shorter RFS (all *p* < 0.05). The RFS of different groups based on DCE, IVIM, and DCE+IVIM prediction models was significantly different (all log-rank *p* < 0.05). The same conclusion was reached in further subgroup analysis in the ypStage II/III, LVI positive, and PNI positive groups. Different studies (40, 41) have shown that pathological TRG grade was a predictor of OS and RFS in LAGC patients. Patients with good response had obvious tumor tissue fibrosis, less tumor residual, and down-staging, which were correlated with better prognosis. DCE-MRI and IVIM-DWI make it possible to predict NCT response before treatment, which can provide a basis for the selection of individualized treatment plans for LAGC patients.

The present study also has several limitations. First, the sample size is relatively small, which requires more cases to be verified, so as to be applied in clinical practice. Second, manually drawing ROI in the slices at the greatest diameter might introduce certain subjectivity of measurement, while 3D voxel-by-voxel analyses might have yielded more reliable and repeatable results for biological tumor heterogeneity. Third, a total of seven patients were excluded due to the inferior image quality. Therefore, the stability of MRI image quality in gastric cancer needs to be further improved. Fourth, the follow-up time was comparatively short (median follow-up time 15.0 months), and the clinical endpoints were not evaluated as OS rate. These limitations need to be addressed in future studies.

## Conclusion

In conclusion, this study demonstrated that pretreatment DCE-MRI quantitative parameters K^trans^, k_ep_, v_e_, and IVIM-DWI parameter D value were independent predictors of NCT response for LAGC. The regression models based on baseline DCE-MRI, IVIM-DWI, and their combination could predict the RFS of patients. This is of great value for clinicians to choose the most appropriate and individualized treatment strategy for LAGC patients.

## Data Availability Statement

The original contributions presented in the study are included in the article/**Supplementary Material**. Further inquiries can be directed to the corresponding author.

## Ethics Statement

The studies involving human participants were reviewed and approved by the Independent Ethics Committee of the Cancer Hospital, Chinese Academy of Medical Sciences (Beijing, China). The patients/participants provided their written informed consent to participate in this study.

## Author Contributions

YJZ and LJ contributed to the conception and design of the study. YJZ, ZJ, BW, and YXZ contributed to the enrollment of the patients. YJZ, ZJ, and BW collected the clinical data and performed followed-ups. YL and JJ performed the MRI examination. YJZ and SW performed the data analysis and interpretation. YJZ wrote the first draft of the manuscript. SW and LJ revised the language and reviewed the manuscript. All authors contributed to the article and approved the submitted version of the manuscript.

## Funding

This work was supported by the Beijing Hope Run Special Fund (No. LC2016A06). The funders played no role in data collection and analysis, design, decision to publish, or preparation of the manuscript.

## Conflict of Interest

Author SW was employed by GE Healthcare, Life Sciences.

The remaining authors declare that the research was conducted in the absence of any commercial or financial relationships that could be construed as a potential conflict of interest.

## Publisher’s Note

All claims expressed in this article are solely those of the authors and do not necessarily represent those of their affiliated organizations, or those of the publisher, the editors and the reviewers. Any product that may be evaluated in this article, or claim that may be made by its manufacturer, is not guaranteed or endorsed by the publisher.
