# Supplementary material for: Quantitative Dynamic-Enhanced MRI and Intravoxel Incoherent Motion Diffusion−Weighted Imaging for Prediction of the Pathological Response to Neoadjuvant Chemotherapy and the Prognosis in Locally Advanced Gastric Cancer
Source: Front Oncol. 2022 Mar 29;12:841460. doi: 10.3389/fonc.2022.841460 (PMC9001840; doi:10.3389/fonc.2022.841460)
Supplement: Supplementary file 1 [file DataSheet_1.docx]

**Supplementary Material**

**Supplementary Table 1.** MR imaging acquisition protocol and main sequence parameters

| Sequence | Plane | TR (ms) | TE (ms) | FOV (mm) | Matrix | FA (degrees) | ST/gap (mm) | NEX | Bandwidth (kHz) | Acquisition time |
| --- | --- | --- | --- | --- | --- | --- | --- | --- | --- | --- |
| LAVA-Flex T1WI | Axial | 5.1 | 1.4 | 400 | 288×224 | 12 | 5/0 | 1 | 166.67 | 14 (s) |
| SS-FSE T2WI | Axial | 1548 | 68 | 400 | 288×288 | 90 | 5/1 | 1 | 83.33 | 50 (s) |
| SS-FSE T2WI | Coronal | 1816 | 68 | 400 | 288×288 | 90 | 4/1 | 1 | 83.33 | 45 (s) |
| SS-FSE T2WI | Sagittal | 1548 | 68 | 400 | 288×288 | 90 | 4/1 | 1 | 83.33 | 40 (s) |
| FS T2WI Propeller | Axial | 8,000-10,000/ | 96-100 | 400 | 320×320 | 90 | 5/1 | 2.5 | 50.0 | 3-4 (min) |
| SS-EPI DWI | Axial | 8000-10,000 | 56-60 | 380 | 128×160 | 90 | 5/1 | 2 | 250 | 1.50 (min) |
| IVIM-DWI^*^ | Axial | 4000-8000 | 56-60 | 380 | 128×160 | 90 | 5/1 | 1, 1, 1, 1, 1, 1, 2, 4, 6, and 6 | 250 | 5-6 (min) |
| Precontrast LAVA-XV T1WI | Axial | 2.9 | 1.4 | 380 | 288×224 | 3, 6, 9, and 12 | 5/0 | 1 | 125 | 6 (s) |
| DCE-perfusion MRI | Axial | 2.9 | 1.4 | 380 | 288×224 | 15 | 5/0 | 1 | 125 | 4-6 (min) |
| Postcontrast LAVA-Flex T1WI | Coronal | 4.2 | 1.9 | 400x36 | 352×256 | 15 | 4/0 | 1 | 200 | 16 (s) |

^*^Acquired during free breathing; 10 b-values were used (0, 10, 20, 40, 100, 200, 400, 800, 1000 and 1200 s/mm^2^).

LAVA-Flex, liver acquisition with volume acceleration; LAVA-XV, liver acquisition with volume acceleration-extended volume; FS, fat suppression; SS-FSE, single-shot fast-recovery fast spin-echo; SS-EPI, Single-shot echo-planar imaging; T1WI, T1-weighted imaging; T2WI, T2-weighted imaging; DWI, diffusion-weighted imaging; DCE, dynamic contrast enhanced; TR, repetition time; TE, echo time; Field of view, FOV; Flip angle, FA; ST, slice thickness; NEX, number of excitations

**Supplementary Table 2.** Inter- and intra-observer reproducibility for IVIM-DWI and DCE-MRI parameters measurement

| Parameter | Inter-observer |  | Intra-observer |
| --- | --- | --- | --- |
|  | ICC (95% CI) |  | ICC (95% CI) |
| K^trans^ | 0.921 (0.883, 0.958) |  | 0.942 (0.925, 0.956) |
| k_ep_ | 0.935 (0.914, 0.965) |  | 0.935 (0.914, 0.947) |
| v_e_ | 0.936 (0.902, 0.957) |  | 0.907 (0.892, 0.920) |
| v_p_ | 0.884 (0.856, 0.903) |  | 0.892 (0.863, 0.911) |
| ADC_standard_ | 0.958 (0.923, 0.969) |  | 0.947 (0.928, 0.966) |
| D | 0.964 (0.952, 0.975) |  | 0.950 (0.926, 0.972) |
| D^*^ | 0.866 (0.837, 0.889) |  | 0.883 (0.845, 0.904) |
| *f* | 0.893 (0.868, 0.920) |  | 0.904 (0.877, 0.942) |

ICC, intraclass correlation coefficient; CI, confidence interval.

K^trans^, volume transfer constant; k_ep_, reflux rate; v_e_, volume fraction of the extravascular extracellular matrix; v_p_, plasma volume fraction; ADC_standard_, standard apparent diffusion coefficient; D, true diffusion coefficient; D^*^, pseudo-diffusion coefficient; *f*, microvascular volume fraction.

**Supplementary Table 3.** Univariate and multivariate analyses of clinical, pathological factors and baseline multiparametric MRI quantitative parameters in prediction for tumor response to NCT

| Variable | Univariate analysis | | |  | Multivariate analysis^*^ | | |
| --- | --- | --- | --- | --- | --- | --- | --- |
|  | Odds ratio | 95% CI | *P* |  | Odds ratio | 95% CI | *P* |
| Clinical factors |  |  |  |  |  |  |  |
| Gender |  |  |  |  |  |  |  |
| Male | 1 (reference) |  |  |  |  |  |  |
| Female | 0.678 | 0.270-1.704 | 0.409 |  |  |  |  |
| Age, years | 1.007 | 0.975-1.040 | 0.677 |  |  |  |  |
| MTD, cm | 0.982 | 0.812-1.186 |  |  |  |  |  |
| Location |  |  |  |  |  |  |  |
| EGJ | 1 (reference) |  |  |  |  |  |  |
| Fundus | 4.773 | 0.514-44.330 | 0.169 |  |  |  |  |
| Body | 0.886 | 0.338-2.322 | 0.806 |  |  |  |  |
| Antrum | 1.432 | 0.581-3.531 | 0.436 |  |  |  |  |
| Whole stomach | 0.764 | 0.253-2.306 | 0.633 |  |  |  |  |
| Surgical approach |  |  |  |  |  |  |  |
| Esophagogastrectomy | 1 (reference) |  |  |  |  |  |  |
| Proximal gastrectomy | 0.933 | 0.261-3.343 | 0.916 |  |  |  |  |
| Distal gastrectomy | 1.348 | 0.524-3.466 | 0.535 |  |  |  |  |
| Total gastrectomy | 0.933 | 0.362-2.406 | 0.886 |  |  |  |  |
| DCE-MRI parameters^**^ |  |  |  |  |  |  |  |
| K^trans^ | 9.334 | 3.531-24.672 | <0.001^***^ |  | 5.300 | 1.470-19.104 | 0.011 |
| k_ep_ | 4.442 | 2.297-8.589 | <0.001^***^ |  | 3.918 | 1.484-10.345 | 0.006 |
| v_e_ | 3.221 | 1.837-5.646 | <0.001^***^ |  | 2.926 | 1.437-5.961 | 0.003 |
| v_p_ | 1.190 | 0.795-1.780 | 0.398 |  |  |  |  |
| IVIM-DWI parameters^**^ |  |  |  |  |  |  |  |
| ADC_standard_ | 0.673 | 0.465-0.973 | 0.036^***^ |  |  |  | NS |
| D | 0.221 | 0.127-0.384 | <0.001^***^ |  | 0.266 | 0.138-0.515 | <0.001 |
| D^*^ | 1.131 | 0.796-1.608 | 0.492 |  |  |  |  |
| *f* | 1.368 | 0.953-1.963 | 0.090 |  |  |  |  |

^*^ Forward stepwise, likelihood ratio method was adapted in multivariate logistic regression analysis.

^**^ Data at univariate and multivariate logistic analyses were transformed into a z normalization.

^***^ Data are statistically from the univariate logistic regression.

K^trans^, volume transfer constant; k_ep_, reflux rate; v_e_, volume fraction of the extravascular extracellular matrix; v_p_, plasma volume fraction; ADC_standard_, standard apparent diffusion coefficient; D, true diffusion coefficient; D^*^, pseudo-diffusion coefficient; *f*, microvascular volume fraction; NS, non-significant; DCE-MRI, dynamic contrast-enhanced MRI; IVIM-DWI, intravoxel incoherent motion diffusion-weighted imaging.


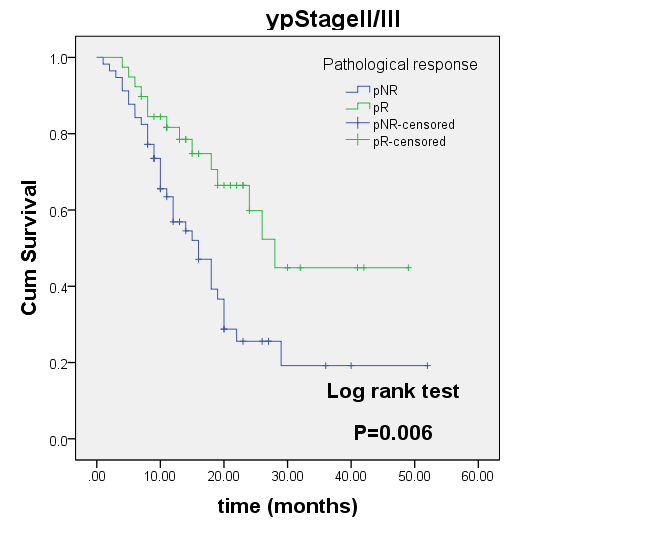

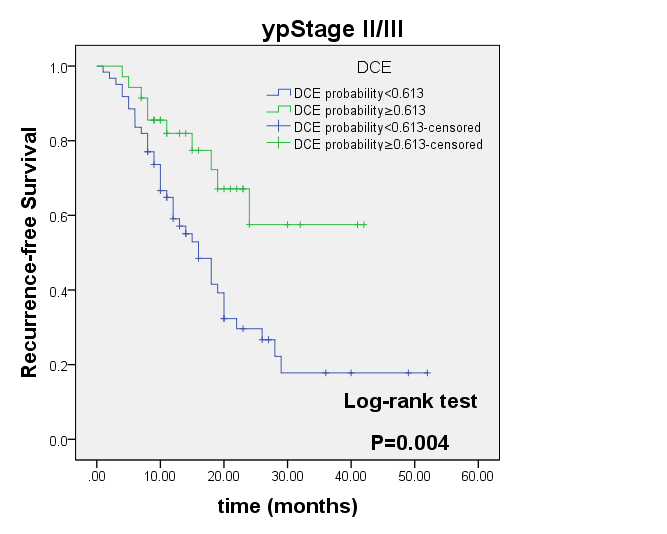

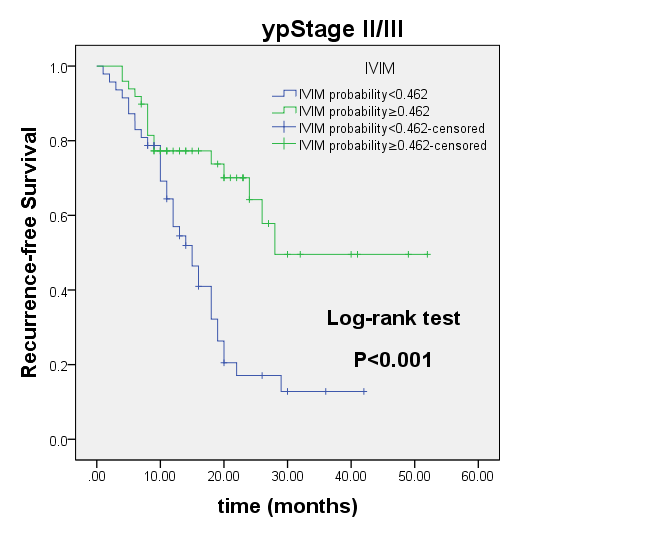

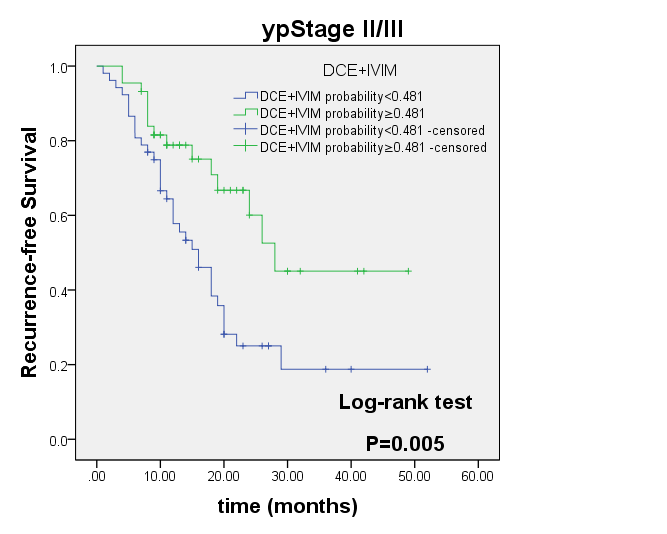

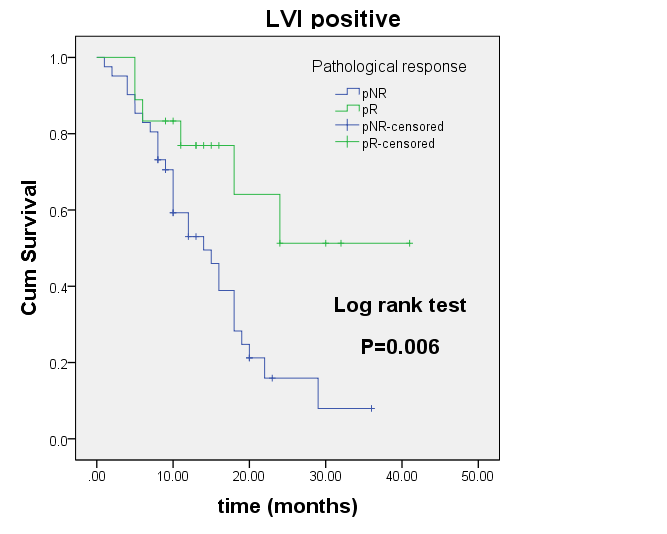

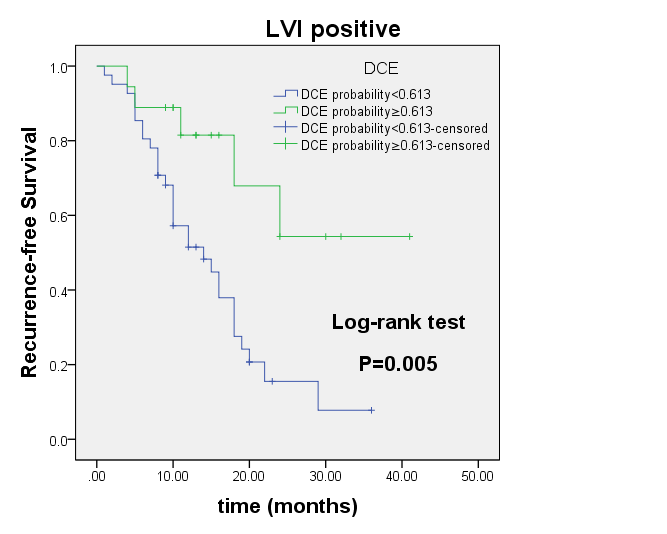

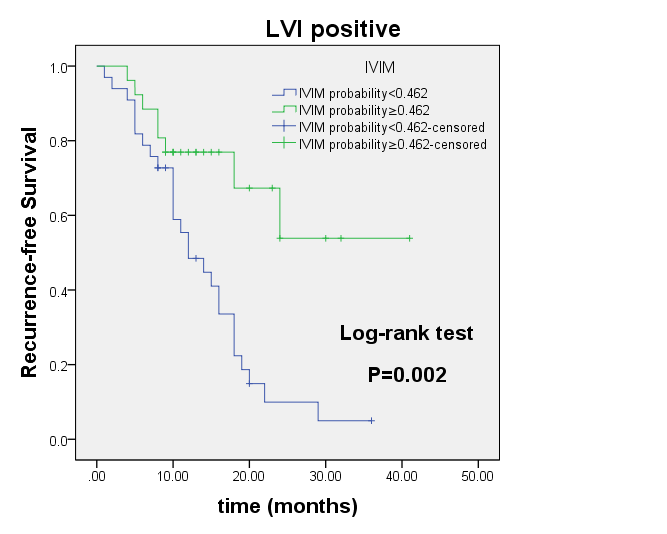

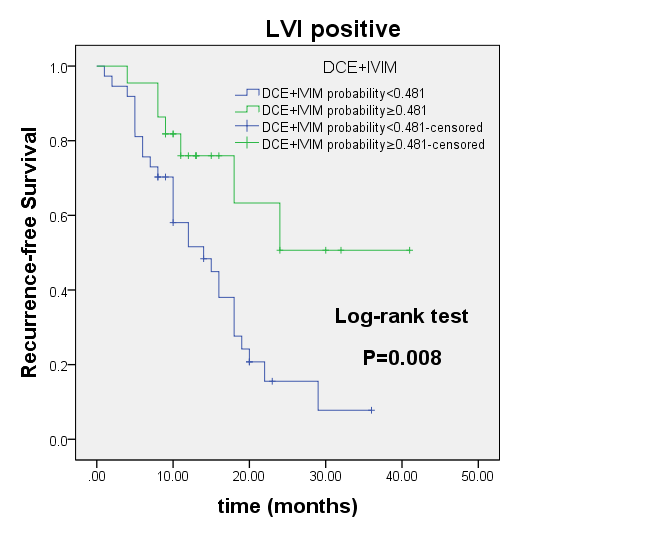

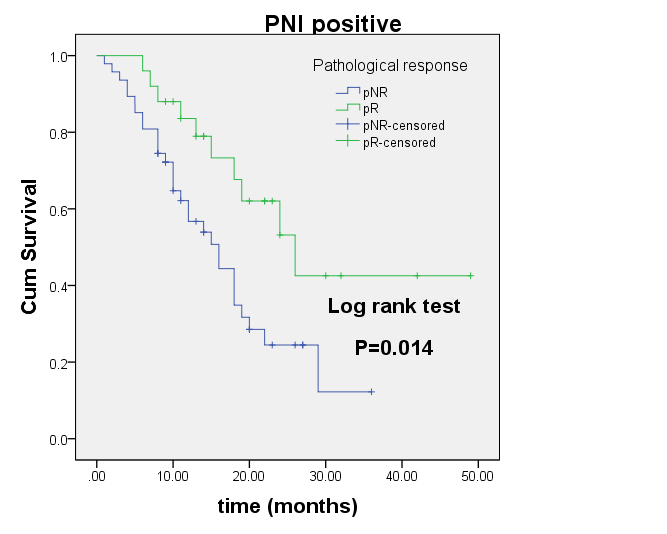

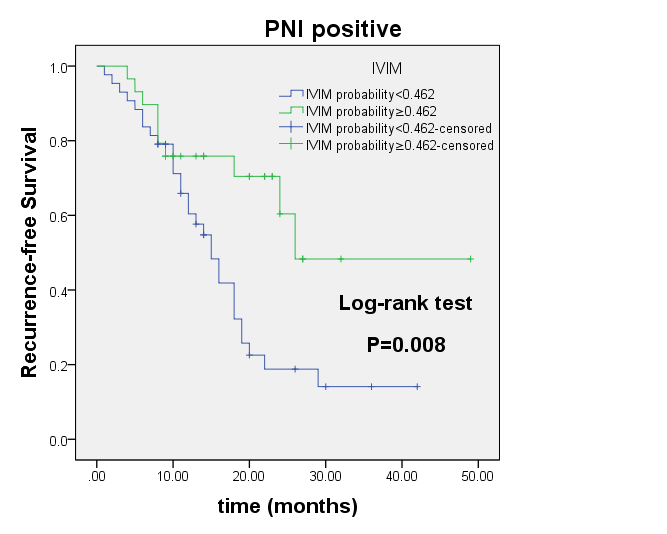

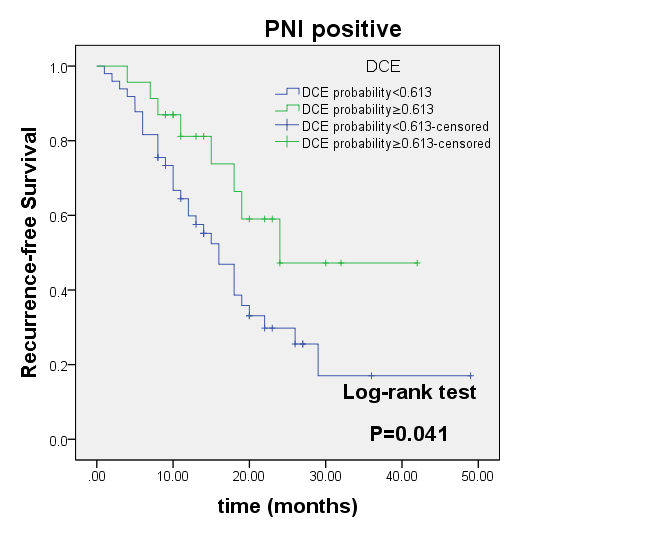

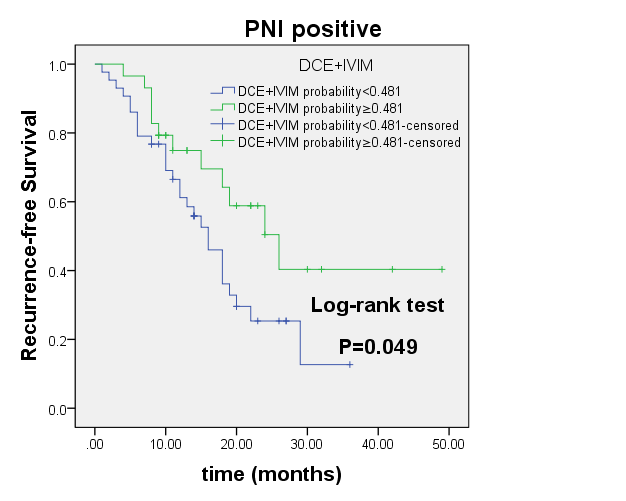


**A**

**B**

**C**

**D**

**E**

**F**

**G**

**H**

**I**

**J**

**K**

**L**

**Supplementary Figure 1.** Subgroup Kaplan-Meier analyses of recurrence-free survival (RFS) in ypStage II/II (n=96) (A-D), lymphovascular invasion positive (n=59) (E-H) and perineural invasion positive (n=72) (I-L). Locally advanced gastric cancer (LAGC) patients stratified by category: responders and non-responders as classified according to pathological response (A, E, I) and predicting models of DCE-MRI (B, F, J), IVIM-DWI (C, G, K) and DCE+IVIM (D, H, L).

DCE-MRI, dynamic contrast-enhanced MRI; IVIM-DWI, intravoxel incoherent motion diffusion-weighted imaging.
